# Calcium Dobesilate Reverses Cognitive Deficits and Anxiety-Like Behaviors in the D-Galactose-Induced Aging Mouse Model through Modulation of Oxidative Stress

**DOI:** 10.3390/antiox10050649

**Published:** 2021-04-23

**Authors:** Elham Hakimizadeh, Mohammad Zamanian, Lydia Giménez-Llort, Clara Sciorati, Marjan Nikbakhtzadeh, Małgorzata Kujawska, Ayat Kaeidi, Jalal Hassanshahi, Iman Fatemi

**Affiliations:** 1 Physiology-Pharmacology Research Center, Research Institute of Basic Medical Sciences, Rafsanjan University of Medical Sciences, Rafsanjan 7717933777, Iran; E.hakimizadeh@rums.ac.ir (E.H.); a.kaeidi@rums.ac.ir (A.K.); hasanshahij@rums.ac.ir (J.H.); 2 School of Nahavand Paramedical, Hamadan University of Medical Sciences, Hamadan 6718773654, Iran; m.zamanian@savehums.ac.ir; 3 Neurophysiology Research Center, Hamadan University of Medical Sciences, Hamadan 6718773654, Iran; 4 Institute of Neuroscience & Department of Psychiatry and Forensic Medicine, Universitat Autònoma de Barcelona, E-08193 Barcelona, Spain; lidia.gimenez@uab.cat; 5 Division of Immunology, Transplantation and Infectious Diseases, IRCCS Ospedale San Raffaele Scientific Institute, 20132 Milan, Italy; sciorati.clara@hsr.it; 6 Department of Physiology, School of Medicine, Tehran University of Medical Sciences, Tehran 14155-6559, Iran; m-nikbakhtzadeh@razi.tums.ac.ir; 7 Department of Toxicology, Poznan University of Medical Sciences, Dojazd 30, 60-631 Poznań, Poland; kujawska@ump.edu.pl; 8 Department of Physiology and Pharmacology, School of Medicine, Rafsanjan University of Medical Sciences, Rafsanjan 7717933777, Iran; 9 Research Center of Tropical and Infectious Diseases, Kerman University of Medical Sciences, Kerman 7616913555, Iran

**Keywords:** aging, antioxidant activity, calcium dobesilate, Doxium, D-galactose, mice, oxidative stress

## Abstract

The long-term treatment of mice with D-galactose (D-gal) induces the overproduction of reactive oxygen species (ROS) and is a well-accepted experimental model of oxidative stress-linked cognitive disorders in physiological aging. Calcium dobesilate (CaD, Doxium^®^) is an established vasoactive and angioprotective drug commonly used for the clinical treatment of diabetic retinopathy and chronic venous insufficiency. It has antioxidant properties and controls vascular permeability. In the current study, we evaluated the protective effects of CaD (50 and 100 mg/kg/day p.o.) in male mice treated with D-gal (500 mg/kg/day p.o.) for six weeks. Results demonstrated that body weight loss, anxiety-like and cognitive impairments of D-gal-treated animals were reversed by CaD administration as evaluated by the measurement of mice performance in elevated plus-maze, Y-maze, and shuttle box tests. CaD treatment also inhibited the oxidative stress in aging mouse brains by decreasing malondialdehyde (MDA) levels and increasing superoxide dismutase (SOD), glutathione peroxidase (GPx), and catalase (CAT) enzyme activities. These results could open new perspectives for the clinical use of CaD in treating and preventing cognitive impairment in older people.

## 1. Introduction

Senescence is the gradual deterioration of cells or organs during aging [1]. The senescence processes have not yet been fully defined, but it is well-documented that oxidative damage plays a crucial rol [2]. Oxidative stress has also been associated with anxiety and cognitive disorders in older people [3]. Overproduction of reactive oxygen species (ROS) ultimately affects the activity of the endogenous antioxidants enzymes, such as superoxide dismutase (SOD), glutathione peroxidase (GPx), and catalase (CAT), reducing their cytoprotective power [4].

Several animal models have been proposed to investigate the mechanisms of aging. Although the “naturally aging model” (the study of the experimental animals over time) may be the most informative to investigate the complexity of aging, it is costly and time-consuming [5]. The need to reduce the analyses’ time and costs led researchers to develop “accelerating aging models”. Among these, the D-galactose (D-gal) model is one of the more affordable because it has few side effects and a high survival rate [6]. Long-term treatment with D-gal in mice reproduces natural oxidative stress in aging through overproduction of ROS, and antioxidant enzyme downregulation [7,8] impacts cognition and behavior [9] and could be employed for anti-aging studies [10]. The dose of D-gal 500 mg/kg allows reproducing oxidative stress and cognitive impairment within six weeks [7,8,10], while lower doses (100 mg/kg) require more prolonged treatment [9]. Male mice are particularly affected by D-gal and are usually employed for antioxidative studies [9].

Calcium dobesilate (CaD) is an established vasoactive and angioprotective drug (Doxium^®^, OM PHARMA, Meyrin, Switzerland) commonly used to treat diabetic retinopathy and chronic venous insufficiency, reducing vascular permeability [11,12]. Previous studies also reported the antioxidant properties of CaD [13,14]. It is a potent free radical scavenger in the retina and reduces the level of malondialdehyde (MDA), inhibiting lipid peroxidation [14]. Moreover, CaD increases the activity of antioxidant enzymes [13,15]. It has been also demonstrated that CaD could reduce cognitive impairment in animal models of Alzheimer’s disease via its anti-inflammatory and antioxidant properties [16].

In the current study, we explored the possible role of CaD against anxiety and cognitive dysfunction as well as against oxidative brain damage induced by D-gal treatment in mice. We found that CaD reversed cognitive deficits and anxiety-like behaviors by reducing oxidative stress.

## 2. Materials and Methods

### 2.1. Animals and Treatments

Twenty-eight 2–3 months old male NMRI (Naval Medical Research Institute) mice (30 ± 2 g) were housed in Plexiglas cages (7 mice per cage) at the Animal House of the Rafsanjan University of Medical Sciences. They were kept at constant temperature (23 ± 2 °C) and humidity (60%) and a 12-h light/dark cycle (lights on at 8:00 a.m.), with free access to food (standard pellet chow; Pars Dam, Tehran, Iran) and water.

Seven days after arrival, the animals were randomly divided into 4 groups (7 animals/group). The control group received standard drinking water (10 mL/kg). D-gal (Sigma-Aldrich, Darmstadt, Germany) and CaD (Doxium^®^, OM PHARMA, Meyrin, Switzerland) were dissolved freshly in drinking water (10 mL/kg) for oral administration. Mice received the drugs daily for 6 weeks. The D-gal group received D-gal alone (500 mg/kg/day) (17,18). The D-gal + CaD 50 group received D-gal plus CaD 50 mg/kg/day, while the D-gal + CaD 100 group received D-gal plus CaD 100 mg/kg/day (17). CaD treatment was conducted without any restriction of food intake, in agreement with the information leaflet provided with its formulation for medical use.

The Guide for the Care and Use of Laboratory Animals (Institute for Laboratory Animal Research, National Research Council, Washington, DC, National Academy Press, no. 85-23, revised 1996) was followed. The Animal Ethics Committee of the Rafsanjan University of Medical Sciences approved this study protocol (Approval ID: IR.RUMS.REC.1397.108).

### 2.2. Body Weight and Behavioral Tests

The physical condition was recorded daily through body weight. Elevated plus-maze (EPM), Y-maze, and shuttle box tests were performed at the end of the drug treatment (day 43 and day 45–47, respectively). All mazes were cleaned with diluted ethanol (5%) between each run.

Animals were acclimated 1 h before the behavioral tests. All the behavioral experiments were done in the morning between 9.00 and 12.00 a.m. All tests were done by a blinded observer.

Twenty-four hours after the last drug administration, the mice were sacrificed (between 12.00 a.m. and 3 p.m.). The order of animals to analyze was randomly determined.

The EPM test was used to evaluate anxiety. This maze consisted of two open (50 × 10 cm) and closed arms (50 × 10 × 40 cm), representing a plus sign. Briefly, the animals were put in the central area of the EPM and observed for 5 min with a digital camera. The percentage of entries (%OAE) and the percentage of time spent into the maze’s open arms (%OAT) were calculated. Locomotor activity (total number of entrances was also measured to confirm no differences in this behavior [17,18].

The Y-maze was used to evaluate the working memory. This maze consisted of three arms (15 × 30 × 40 cm with equal angles between arms). Briefly, the animals were put in the maze center and observed for 8 min with a digital camera. Correct spontaneous alternation of arms, defined as not revisiting the same arm twice before visiting another, was measured. The percentage of correct alternations was calculated as the index: the number of alternations/total arm visits (minus 2) × 100 [19].

The shuttle box was used to evaluate passive avoidance learning and memory. Briefly, the shuttle box had two equal-sized compartments (dark/lit apparatus) with a guillotine door that separated each compartment. This memory test was performed over 3 days. On the first day, the mice were placed into the lit compartment and allowed to move around for 5 min to become habituated to the place. On the second day, the animals were placed in the lit compartment, and after 60 s, the door was opened. When the mice entered the dark compartment, the door was closed, and a mild electrical shock (0.5 mA for 2 s) was delivered to the grid floor. After 20 s, the animals were removed from the dark compartment and returned to its cage. On the third day, the mice were again placed in the light compartment, and the number of shocks before entering the dark compartment was measured (cut-off time was considered 300 s) [20].

### 2.3. Oxidative Stress Evaluation

Twenty-four hours after behavioral tests, mice of each group were sacrificed. Brains were immediately removed, and one hemisphere was homogenized (1/10 *w/v*) in ice-cold Tris-HCl buffer (100 mM, pH 7.4), centrifuged 4427× *g* for 20 min, and the supernatant was collected and stored at −80 °C for the biochemical analyses.

The lipid peroxidation was measured with MDA levels by a commercially available kit (ZellBio, Lonsee, Germany; Catalog Number: ZB-MDA-96A) according to the manufacturer’s protocol (the detection limit: 0.1 µM and the detection range: 0.78–50 µM).

CAT activity was measured by a commercially available kit (ZellBio, Germany; Catalog Number: ZB-CAT-96A), according to the manufacturer’s protocol at a wavelength of 405 nm (the detection limit: 0.5 U/mL and the detection range: 1–100 U/mL).

SOD activity was measured by a commercially available kit (ZellBio, Germany; Catalog Number: ZB-SOD-96A), according to the manufacturer’s protocol (the detection limit: 1 U/mL and the detection range: 5–100 U/mL).

GPx activity was evaluated by a commercially available kit (ZellBio, Germany; Catalog Number: ZB-GPX-96A), according to the manufacturer’s protocol (the detection limit: 5 U/mL and the detection range: 20–500 U/mL).

The light absorption was read by the ELISA Microplate Reader (Rayto, Shenzhen Guangdong, China). All samples were analyzed in duplicate, and the results were presented as a percentage of the control.

### 2.4. Statistical Analysis

Statistical analysis was done by GraphPad Prism software version 6.01 for Windows (San Diego, CA USA). Data were presented as mean ± SEM. For parametric variables, the differences between the groups were analyzed by one-way analysis of variance (ANOVA), followed by the Tukey post hoc test. For non-parametric variables, the groups’ differences were analyzed with Kruskal–Wallis followed by the Dunn post hoc test. A two-way repeated-measures ANOVA (RMA) was used for comparison of body weight changes among experimental groups. Differences were considered statistically significant when *p* < 0.05.

## 3. Results

### 3.1. CaD Inhibited the Body Weight Loss Induced by D-Gal Treatment

**Figure 1:**
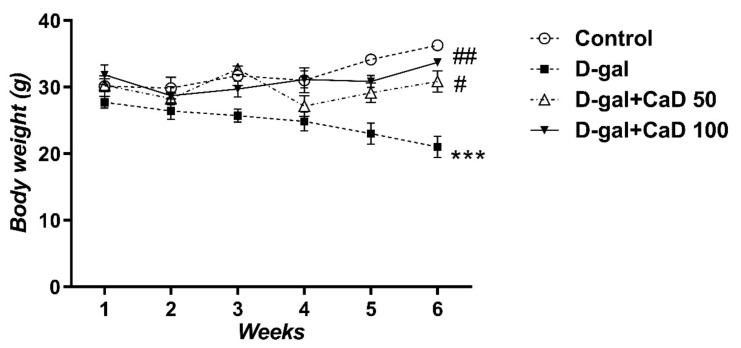
Effect of calcium dobesilate (50 and 100 mg/kg, p.o.) on body weight in D-galactose (D-gal treated) mice. Values are mean ± SEM (*n* = 7 in each group). *** *p* < 0.001 compared to control group; # *p* < 0.05 and ## *p* < 0.01 compared to D-gal treated group. Values are mean ± SEM (*n* = 7 in each group).

D-gal administration decreased the body weight of treated mice compared to the control untreated group (RMA; F (15, 90) = 8.339; *p* < 0.001) (Figure 1). The CaD treatment inhibited the D-gal-induced loss of body weight. The effect was similar in the low dose-treated group (D-gal+CaD 50) and high-dose treated mice (D-gal+CaD 100) (Figure 1, *p* < 0.05 and *p* < 0.01, respectively).

### 3.2. CaD Decreased the Anxiety-Like Behavior in D-Gal Treated Mice

**Figure 2:**
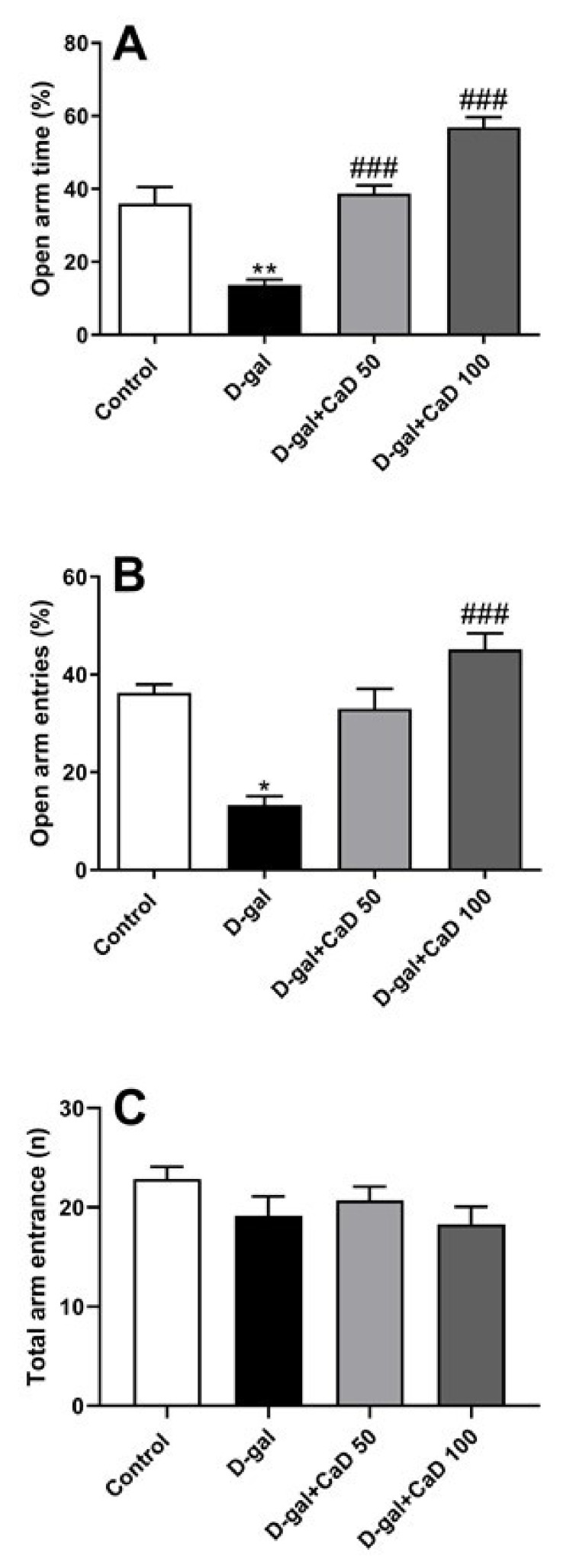
Effect of calcium dobesilate (CaD; 50 and 100 mg/kg, p.o.) in control of mice anxiety-like behavior induced by D-gal. Behavior was evaluated by elevated plus-maze. (**A**) Open arm time, (**B**) Open arm entries and (**C**) Total arm entrance. Values are mean ± SEM (*n* = 7 in each group). * *p* < 0.05 and ** *p* < 0.01 as compared to control group; ### *p* < 0.001 as compared to D-gal treated group.

The development of anxiety-like behavior was evaluated in D-gal treated mice by the EPM test. The results revealed that administration of D-gal decreased the time spent in the open arms (%OAT) (F (3, 24) = 35.31; *p* < 0.01) and percentage of entries into open arms (%OAE) (F = 18.48; *p* < 0.05) compared to the control untreated mice (Figure 2A,B, respectively). CaD administration at the doses of 50 mg/kg in association with D-gal treatment increased %OAT (*p* < 0.001) compared to the D-gal treatment. Treatment with 100 mg/kg CaD increased %OAT (*p* < 0.001) and %OAE (all *p* < 0.001) in comparison with the D-gal-treated animals. No statistically significant changes were found in the locomotor activity (evaluated as the number of entries into the arms) between the various experimental groups (F = 3.441, all *p* > 0.05) (Figure 2C).

### 3.3. CaD Attenuated the Cognitive Impairments in D-Gal Treated Mice

**Figure 3:**
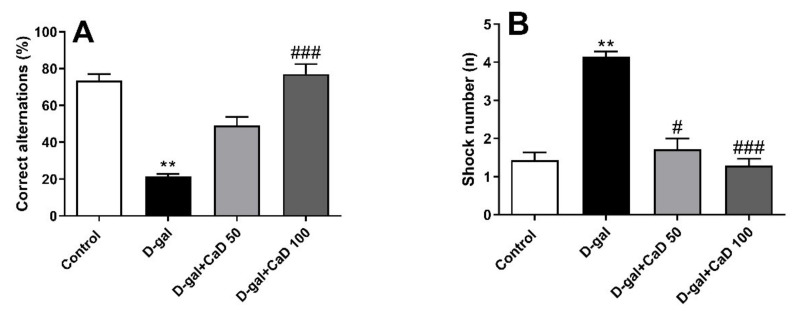
Effect of calcium dobesilate (CaD; 50 and 100 mg/kg, p.o.) on Y-maze (**A**) and shuttle box (**B**) in D-galactose (D-gal) treated mice. Values are mean ± SEM (*n* = 7 in each group). ** *p* < 0.01 as compared to control group; # *p* < 0.05 and ### *p* < 0.001 as compared to D-gal treated group.

The D-gal treated mice showed impaired working memory in comparison with the control group as evaluated by the Y-maze test (F = 21.58; *p* < 0.01) (Figure 3A). CaD at the dose of 100 mg/kg reversed the D-gal effects on working memory, as evidenced by a higher percentage of correct alternations (*p* < 0.001). The D-gal administered mice also showed impaired passive avoidance memory compared to the control group (F = 17.82; *p* < 0.01) as evaluated by the shuttle box test (Figure 3B). CaD at the doses of 50 and 100 mg/kg improved the passive avoidance memory compared to the D-gal group (*p* < 0.05 and *p* < 0.001, respectively).

### 3.4. CaD Reduced the Oxidative Stress s in D-Gal Treated Mice

**Figure 4:**
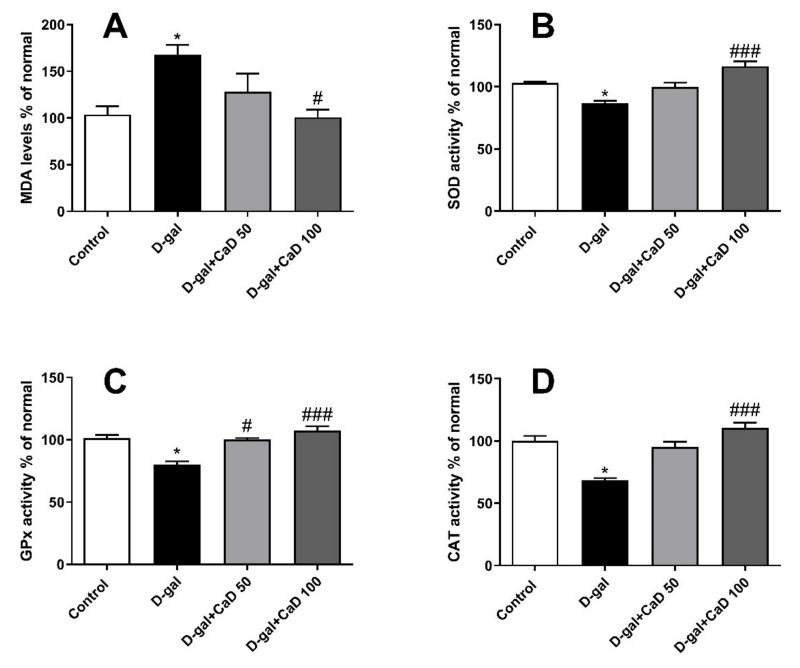
Effect of calcium dobesilate (CaD; 50 and 100 mg/kg, p.o.) on oxidative stress indices in D-gal treated mice. (**A**) MDA levels % of normal, (**B**) SOD activity % of normal, (**C**) GPX activity % of normal, (**D**) CAT activity % of normal. Values are mean ± SEM (*n* = 7 in each group). * *p* < 0.05 as compared to control group; # *p* < 0.05 and ### *p* < 0.001 as compared to D-Gal treated group; MDA: malondialdehyde; SOD: superoxide dismutase; GPx: glutathione peroxidase; CAT: catalase.

Oxidative stress was evaluated by measuring MDA level and evaluating SOD, GPx, and CAT activity in brain homogenates. MDA level in D-gal administered mice was significantly higher in comparison to control animals (F = 10.44; *p* < 0.05) (Figure 4A). CaD at the dose of 100 mg/kg mitigated the increase in MDA levels in D-gal treated animals (*p* < 0.05).

The SOD activity was significantly decreased in the D-gal group in comparison to the control animals (F = 18.13; *p* < 0.05) (Figure 4B). Interestingly, treatment with CaD at the dose of 100 mg/kg increased the SOD activity compared to the D-gal group (all *p* < 0.001). The activity of GPx was significantly decreased in the D-gal group in comparison to the control group (F = 17.60; *p* < 0.05) (Figure 4C). Administration of CaD (50 and 100 mg/kg for 6 weeks) significantly increased GPx activity compared to the D-gal group (*p* < 0.05 and *p* < 0.001, respectively). Administration of D-gal resulted in a remarkable decrease in brain CAT activity when compared to the control group (F = 17.10; *p* < 0.05) (Figure 4D). Administration of CaD (100 mg/kg) to D-gal administered mice increased the brain CAT activity when compared with the D-gal group (*p* < 0.001).

## 4. Discussion

D-gal administration to mice is an established experimental model of aging-linked cognitive and behavior disorders and can be employed to study anti-aging drugs [5]. D-gal treated animals exhibited enhanced brain oxidative stress demonstrated to be involved in cognitive and behaviors abnormalities [5,21]. The current study demonstrated that CaD attenuated cognitive and behavioral impairments induced by D-gal through the control of lipid peroxidation and oxidative stress in brain tissue.

Anxiety disorders are a common comorbidity among older adults [22]. It is well documented that D-gal increases anxiety-like behavior in animals [9,23]. In the current study, we employed three different paradigms to assess the increase in anxiety-like behaviors following the D-gal treatment, and we demonstrated that CaD was able to reduce these behaviors. It has been reported that D-gal-induced anxiety could be linked to an increase in oxidative stress and a decrease in the activity of antioxidant enzymes in the brain [23]. Previous reports have also demonstrated that the administration of antioxidants, such as resveratrol and ascorbic acid, attenuates acute stress-induced anxiety by increasing the activity of antioxidant enzymes, such as SOD, GPx, and CAT [24,25,26]. CaD has previously been shown to mitigate oxidative stress during cardiac surgery [27] and increase the activity of antioxidant enzymes, such as SOD and GPx [13,27,28,29]. Here, we demonstrated that the administration of CaD, an angiopreventive drug clinically approved for the treatment of diabetic retinopathy [30], inhibited oxidative stress as revealed by decreased MDA levels and the increased activity of the antioxidative enzymes SOD, GPx, and CAT in brain homogenates.

Chronic administration of D-gal in animals has deleterious effects on cognitive performance [17,31] and may mimic the decline in cognitive function that accompanies aging [21]. Accordingly, the present results show that working memory and learning and memory of passive avoidance were impaired in the D-gal group of mice. Our results also demonstrated that CaD attenuated these deleterious effects of D-gal. Antioxidant agents and compounds that increase the antioxidant system capacity improve cognition impairment in aging animals and humans [17,31,32,33]. Furthermore, CaD mitigates cognitive disorders in an Alzheimer’s disease model in mice by inhibiting the inflammatory process [16].

It is well established that ROS production increases with age [34] and promotes age-related degenerative processes [35]. In the present work, we demonstrated that CaD was a potent radical scavenger and increased antioxidant enzymatic activity in the brain. This observation has already been suggested in many other pathological conditions. For instance, in ischemia–reperfusion mouse models and diabetic rats, CaD exerts protective effects against intestinal injury by increasing the total antioxidant capacity [36,37]. Similarly, during coronary artery bypass, CaD exerts its beneficial effects by increasing the activity of SOD and GPx and decreasing the level of MDA [27]. In another study, CaD inhibits gentamicin-induced nephrotoxicity by increasing the reducing/antioxidant tissue ferric potency [29]. Finally, a recent study established that CaD has beneficial effects against D-gal-induced cataracts in rats by stabilizing the oxidant/antioxidant balance of the lens and improving the signaling of Nrf2-Keap1, a key regulator of oxidative status in vivo [38]. The present results demonstrated that CaD reduced cerebral oxidative stress, as revealed from its effects on MDA levels, as well as SOD, GPx, and CAT activity, suggesting the potential therapeutic use of CaD as an antioxidant/protective drug in the central nervous system. Of note, in this study, we used male mice because D-gal induces higher oxidative stress in this sex, as we have previously demonstrated [9]. Female mice’s brain is characterized by lower oxidative stress and greater antioxidant capacity [39,40]. Further studies should investigate sex-dependent differences to evaluate, for example, the role of the estrous cycle.

Among the mediators involved in brain oxidative stress is the highly reactive free radical NO, which exerts key functions in the brain [41]. For a long time, it has been described as a “Janus“ molecule with both neuroprotective and neurotoxic effects [42]. In particular, NO concentrations change in the hippocampus during physiological and pathological aging (in Alzheimer’s disease) and are linked to functional impairment [43]. Moreover, nitrosative stress is involved in D-galactose-induced neuronal damage in the cerebellum [44]. The NO levels were increased in the cerebellum of D-galactose-treated rats and contributed to oxidative load leading to protein damage. In this model, antioxidant compounds, such as curcumin and piperine, decreased NO levels, reducing neurotoxicity and degeneration. NO involvement in the mechanism of action of CaD could be hypothesized. Although CaD has been described to enhance NO synthase in the endothelium [45], CaD acts as an antioxidant and may play a role in the neuromodulation of brain functions in aging and neurodegenerative diseases through both stress mechanisms [39].

Previous reports show that the brain–blood barrier (BBB) breakdown and permeability increase occur in both natural [46], D-gal-induced aging [47], and other age-related neuropathological conditions [48]. Under physiological conditions, CaD does not cross the blood barriers of the body. However, as demonstrated in diabetic retinopathy [30], the retina blood barrier breakdown allows the entry of CaD, which protects retina neurons [49]. Although we did not assess the permeability of BBB in our experimental conditions, increased blood–brain barrier permeability in D-gal-treated mice [47] might allow the entry of CaD. On the other hand, CaD could decrease damaged vessels’ permeability and reduce the entry of inflammatory mediators and ROS into the brain tissue [50]. CaD has previously been shown to improve cognitive function via anti-inflammatory and antioxidant effects in a mouse model of Alzheimer’s disease [16]. The D-gal treatment induces significant neuroinflammation with a marked increase in hippocampus inflammatory cytokines, such as TNF-α, IL-6, and IL-1β and astrocytes activation, which could induce neuronal death [5]. Although we did not investigate brain inflammation and neuronal degeneration, our data demonstrated that CaD controlled oxidative stress in the brain. This effect could quench the inflammatory cascade and neurodegeneration enhanced by oxidative stress in D-gal treated mice.

In agreement with previous studies [9,51], we demonstrated that the D-gal-induced aging model resulted in a significant reduction in body weight as compared to controls. Interestingly, we observed that both low and high doses of CaD blocked this effect.

Since CaD has already been clinically approved for treating micro-vascular damage-related diseases, such as diabetic retinopathy and nephropathy, and it has few side effects [12], it could represent an interesting new tool to prevent oxidative damage in aging.

## 5. Conclusions

The current study demonstrated that CaD mitigated anxiety and cognitive-behavioral disorders in the D-gal-induced aging experimental model. CaD reduced the oxidative stress by increasing SOD, GPx, and CAT activities and decreased MDA levels to protect against D-gal detrimental effects. Despite the limitations of the D-gal-induced aging model, our results suggest that CaD, which is already approved for clinical use and safe, could be an interesting pharmacological tool to mitigate or prevent age-related neurological dysfunction.

## Data Availability

The data presented in this study are available on request from the corresponding author.
